# Melatonin promotes the proliferation of GC-1 spg cells by inducing metallothionein-2 expression through ERK1/2 signaling pathway activation

**DOI:** 10.18632/oncotarget.20019

**Published:** 2017-08-07

**Authors:** Chunjin Li, Xiaoling Zhu, Shuxiong Chen, Lu Chen, Yun Zhao, Yanwen Jiang, Shan Gao, Fengge Wang, Zhuo Liu, Rong Fan, Liting Sun, Xu Zhou

**Affiliations:** ^1^ College of Animal Sciences, Jilin Provincial Key Laboratory of Animal Embryo Engineering, Jilin University, Changchun, Jilin, 130062, P.R. China

**Keywords:** GC-1 spg cell proliferation, melatonin, metallothionein-2, transcriptome sequencing

## Abstract

Synthesized by the pineal gland, melatonin is a neurohormone implicated in diverse physiological functions via several mechanisms. However, the role of melatonin in spermatogenesis and its underlying mechanisms have yet to be completely understood. In the present study, transcriptome sequencing was performed to characterize the mechanism of melatonin-induced GC-1 spg proliferation. Gene ontology (GO) enrichment and pathway analyses were also conducted to identify the signaling pathways and biological processes involved in differential mRNA expression. Results revealed 28 differential genes. Of these genes, 11 were upregulated and 17 were downregulated. Melatonin increased the expression of metallothionein-2 (*Mt2*), a gene that acts as a protector to sequester nonessential toxic heavy metals. Functional investigations demonstrated that *Mt2* overexpression promoted the proliferation of GC-1 spg cells, but *Mt2* knockdown significantly suppressed their proliferation and increased their apoptosis. Mechanistic analysis indicated that the extracellular-signal-regulated kinase 1/2 (ERK1/2) pathway participated in melatonin-promoted proliferation of GC-1 spg cells. Therefore, melatonin induces the proliferation of GC-spg 1 cells by stimulating *Mt2* expression, and this process is mediated by the ERK1/2 signaling pathway.

## INTRODUCTION

Male gametes are produced during spermatogenesis occurring in seminiferous tubules inside the testis. Spermatogenesis is a complex process through which spermatogonial stem cells (SSCs) differentiate into spermatozoa. This process involves three phases: mitotic phase (spermatogonial proliferation and differentiation), meiotic phase (chromosomal reduction in spermatocytes via meiosis), and spermatid differentiation into spermatozoa [1]. The successful differentiation of primordial germ cells into mature spermatozoa is mainly controlled by hormones released by the hypothalamic–pituitary–testis axis and growth factors [2]. In addition to the hypothalamic–pituitary–testis axis, the pineal gland is essential for male reproduction [3, 4].

Melatonin is the main neurohormone secreted by the pineal gland, and this hormone is involved in diverse physiological functions, such as male reproduction [5]. In male reproduction, melatonin plays regulatory roles in the testicular development of seasonal breeding animals and affects the release of gonadotropins, which regulate spermatogenesis and testosterone synthesis [6]. Melatonin as a potent free radical scavenger influences sperm quality [7, 8] and performs testicular regulatory roles possibly mediated by receptors in testicular tissues of different species [9, 10]. This hormone can also alter the expression profile of miRNAs in mouse testis, as indicated by indirect evidence [11]. The regulatory roles of melatonin in testicular cells have been further investigated in detail. Sertoli cells play an essential role in spermatogenesis regulation by providing physical support for germ cells and thus form the blood–testis barrier and secrete protein products [12]. Sertoli cells are sensitive to hormonal actions, and the proliferation and metabolism of these cells are regulated by hormones [13]. For example, bovine Sertoli cells contain melatonin receptors 1 and 2, and the expression of these receptors and spermatogenesis-regulated genes is influenced by melatonin in a time- and dose-dependent manner [14]. The metabolites of Sertoli cells are also vital for germ cell survival, and the metabolism of Sertoli cells may be mediated by hormones, such as estrogen and androgen [13]. In an *in vitro* model, the glycolytic profile of Sertoli cells can be altered by melatonin [15]. Thus, spermatogenesis may be affected by melatonin through the regulation of Sertoli cell functions. For instance, spermatogenesis successfully occurs through the renewal and differentiation of spermatogonial stem cells [16]. To confirm the role of melatonin in spermatogenesis, researchers cultured dissociated cells and seminiferous tubular fragments from sheep testis for 30 days and found that melatonin significantly increases testosterone concentrations and expression of luteinizing hormone receptor (LHR) and steroidogenic acute regulatory protein (StAR) [17]. Melatonin also significantly promotes the differentiation of cultured SSCs into haploid germ cells; therefore, melatonin is implicated in spermatogenesis [17]. Our previous study provided direct evidence showing that melatonin promotes the proliferation of GC-1 spg cells by downregulating miR-16 expression [11].

Melatonin elicits beneficial effects on male reproduction. For instance, this hormone protects the testis from oxidative damage and promotes germ cell growth. However, relevant molecular mechanisms remain unknown. Various molecular mechanisms involved in spermatogenesis have been identified through different methods [18, 19], and some of these mechanisms are regulated by different signaling pathways, including phosphatidylinositol 3-kinase (PI3K), extracellular signal–regulated kinases 1 and 2 (ERK1/2), and transforming growth factor-beta (TGF-beta) pathways [20–22].

Although studies have revealed that exposure to melatonin alters the microRNA expression in the testis, studies have yet to determine whether melatonin can regulate the changes in transcriptome in germ cells. Melatonin-regulated cellular functions, including growth and apoptosis [23, 24], involve ERK1/2 signaling pathway. Nevertheless, the interaction of melatonin and ERK1/2 signaling pathway in germ cells has yet to be examined in detail. To obtain further insights into melatonin-promoted proliferation of GC-1 spg cells, we investigated melatonin-regulated genes through transcriptome sequencing and observed the interaction of melatonin and ERK1/2 signaling pathway.

## RESULTS

**Figure 1:**
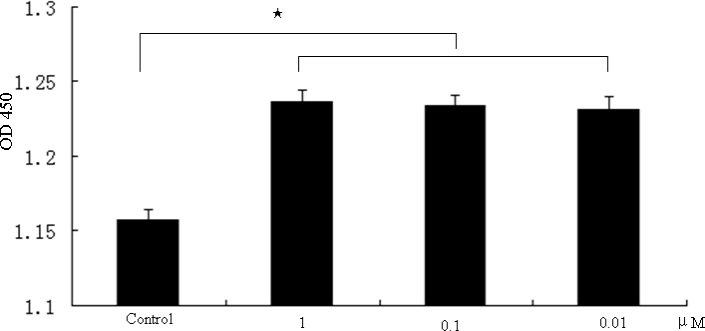
Promotion of cell proliferation by melatonin in GC-1 spg cells GC-1 spg cells were treated with different concentrations of melatonin (1–0.01 μM). Data represent the mean ± SD of the results in three independent experiments. ★ indicates significant differences (*p* < 0.05).

The effect of melatonin on the proliferation of GC-1 spg cells was determined with CCK8 assays after these cells were treated with different melatonin concentrations (1–0.01 μM) for 24 hrs. Consistent with previous findings [11], our results confirmed that melatonin induces a statistically beneficial effect on the proliferation of GC-1 spg cells (Figure 1). Melatonin of 10nM was used for the follow-up experiments.

**Figure 2:**
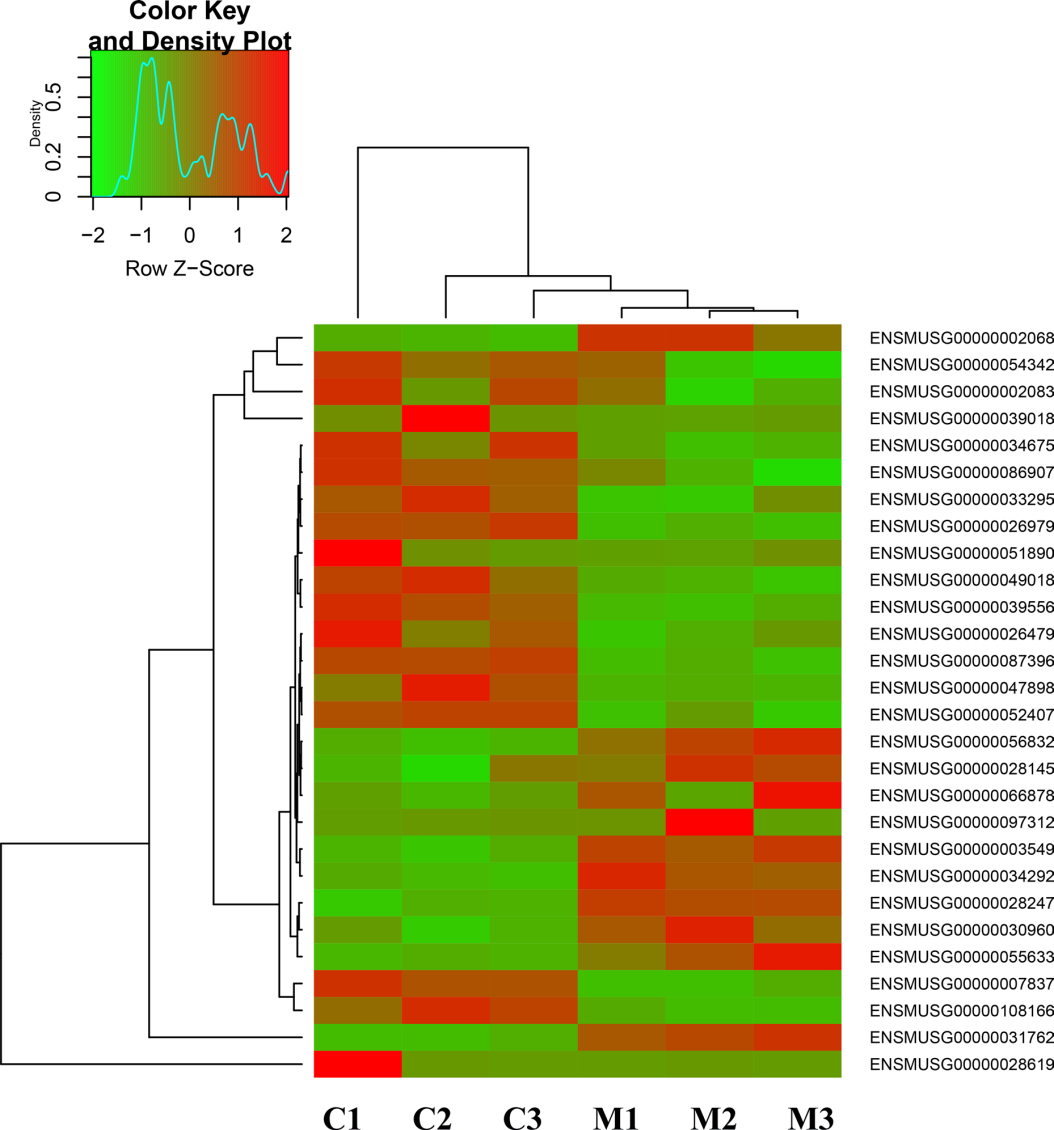
The distinct expression patterns of transcriptome in melatonin-regulated GC-1 spg cells Hierarchical clustering of transcriptome that showed the different gene expression profiles in GC-1 spg cells after melatonin treatment. Expression levels are defined by the color key on the top-left side of the figure. C indicates control, M indicates melatonin treatment.

In the current experiment, transcriptome sequencing arrays were used to determine the mRNA expression profile of GC-1 spg cells induced by melatonin, which could account for the significant proliferation effect. After 24 hrs stimulation on GC-1 spg cell, different patterns of mRNA expression were observed (Figure 2). RNA sequencing alignments revealed 18,526 genes. Of these genes, 11 were upregulated and 17 were downregulated. These 28 genes were differentially expressed between melatonin-stimulated and control groups (Supplementary Table 1).

**Figure 3:**
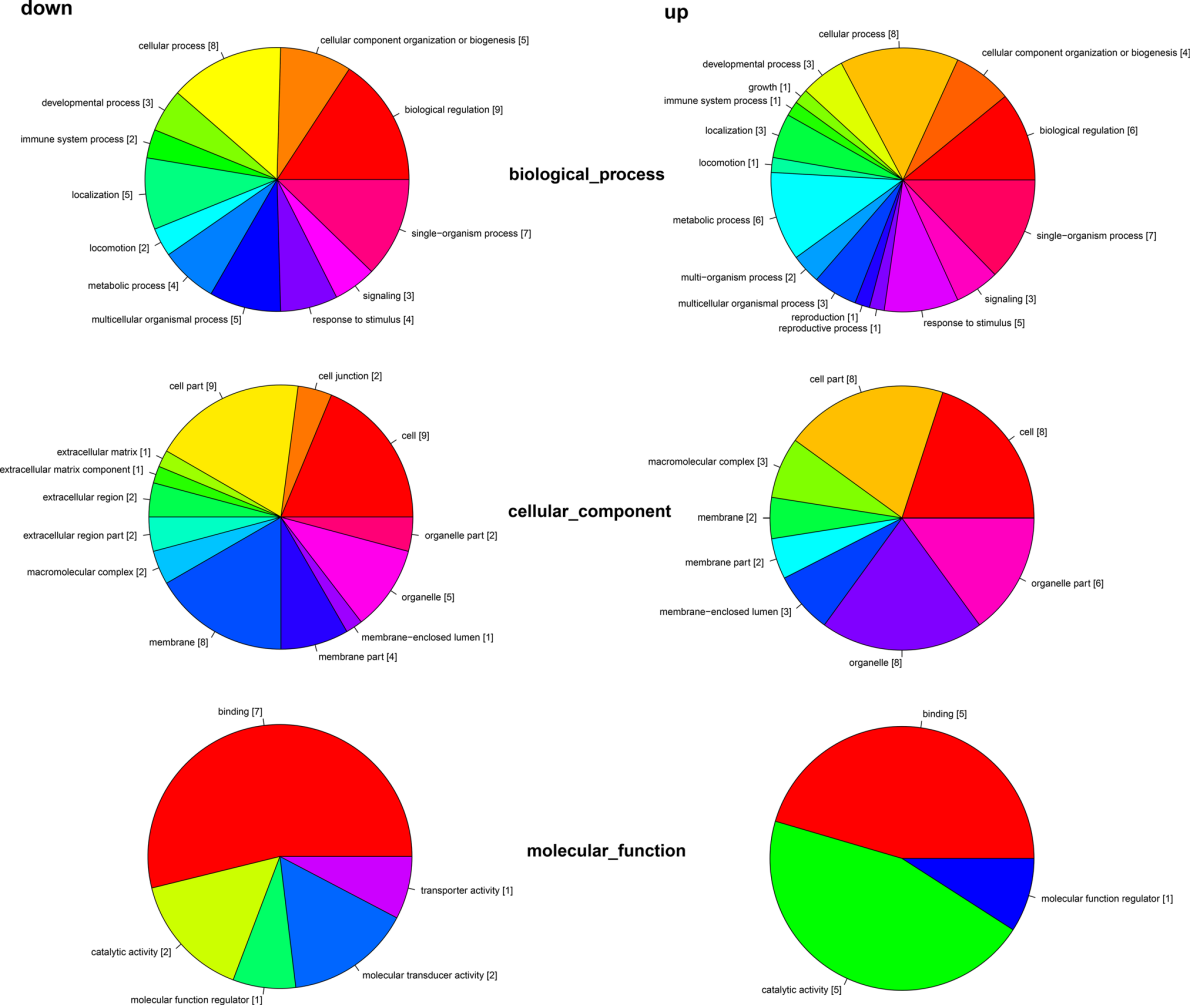
Distribution of transcriptome by gene ontology (GO) All sequences with Blast2GO matches were assigned to three GO categories (biological process, cellular component, and molecular function) and classified into 34 functional terms. Data are shown for both the downregulated (left) and upregulated (right) transcriptome regulated by melatonin.

The differentially expressed genes were classified into 3 GO categories and 34 terms through gene ontology analysis (Figure 3). In the biological process category, the differentially expressed genes were classified into 16 terms, including cellular process, biological regulation, growth, reproduction, and et al. In the cellular component categories, the differentially expressed genes were classified into 13 terms, including cell part, organelle part, membrane, macromolecular complex, and et al. In the molecular function category, the differentially expressed genes were classified into 5 terms, including binding, transporter activity, molecular transducer activity, molecular function regulator, catalytic activity, and et al.

**Figure 4:**
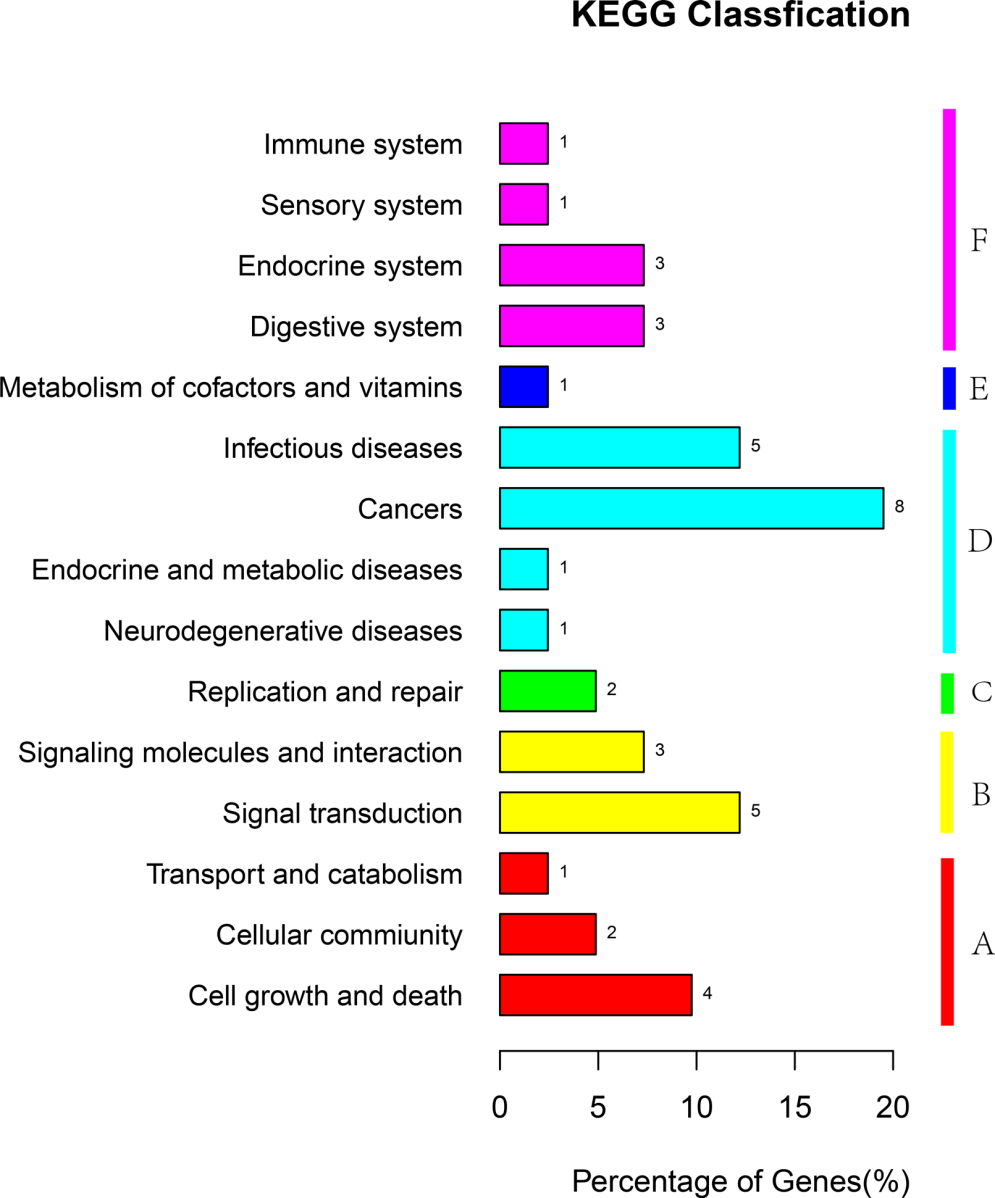
KEGG classification of unigenes The unigenes were summarized into six main categories (a: cellular processes, b: environmental information processing, c: genetic information processing, d: human diseases, e: metabolism, and f: organismal systems,). The x-axis represents the unigenes’ respective categories, whereas the y-axis represents the percentage of unigenes.

The differentially expressed genes were compared with the KEGG database using BLASTX, and the corresponding pathways were established. Among the differentially expressed genes assigned to the KEGG pathway 1, 2, 8, 7, 8 and 15 were assigned to metabolism, genetic information, environmental information processing, cellular process, organismal systems, and human diseases, respectively (Figure 4). These annotations provided valuable information to investigate specific processes, functions, and pathways influencing the role of melatonin in male reproduction.

**Figure 5:**
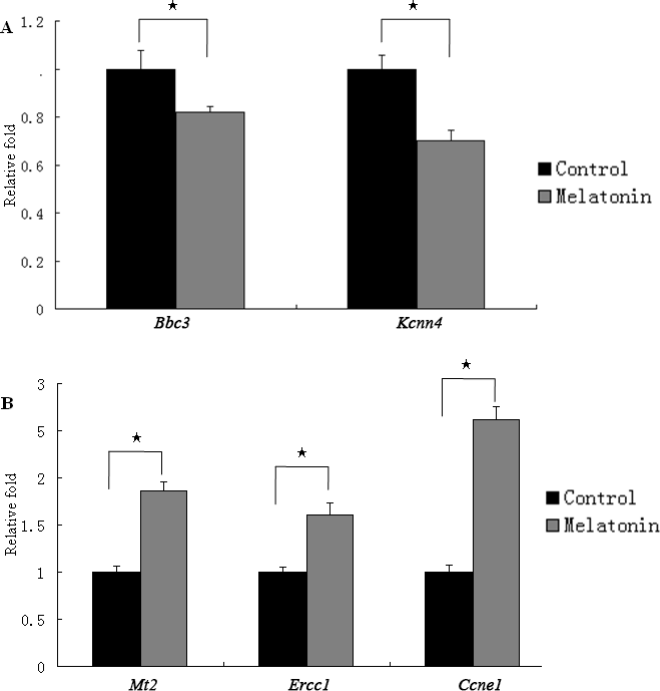
The expression of genes regulated by melatonin in GC-1 spg cells Five differentially expressed genes identified by transcriptome sequencing were determined using qRT-PCR. The mRNA level of *Bbc3* and *Kcnn4* was downregulated by melatonin (**A**). The mRNA level of *Mt2, Ercc1,* and *Ccne1* was upregulated by melatonin (**B**). ★ indicates significant differences (*p* < 0.05).

The differentially expressed mRNAs were subjected real-time quantitative PCR to validate the mRNA sequencing data. Five genes, namely, two downregulated and three upregulated genes, associated with DNA replication and repair and cell cycle were quantified. Real-time quantitative PCR data were consistent with mRNA sequencing results (Figure 5). The *Mt2* expression was significantly increased after the GC-1 spg cells were treated with melatonin.

**Figure 6:**
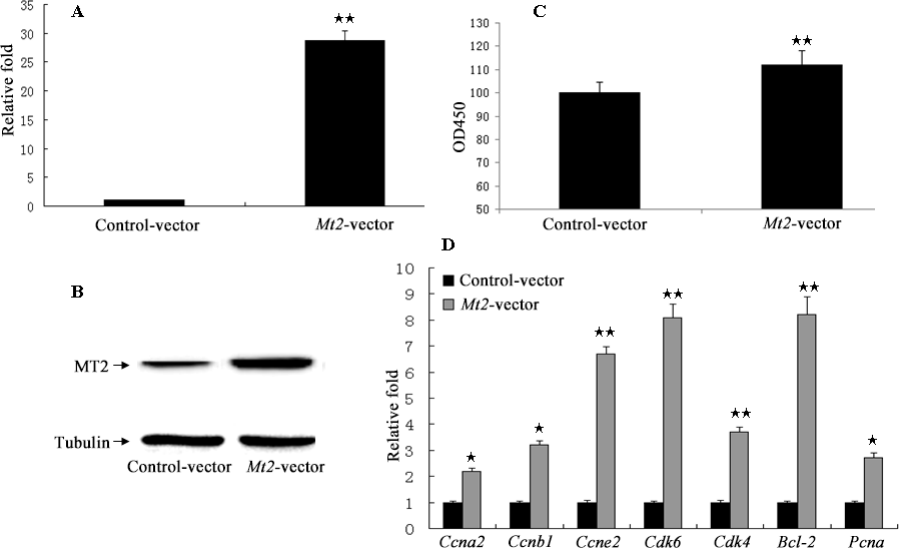
The cell proliferation assay after *Mt2* overexpression in GC-1 spg cells Overexpression plasmid of *Mt2* was constructed using pCMV6 plasmid. Plasmid was transfected into GC-1 spg cells with Lipofectamine 2000. Both mRNA (**A**) and protein (**B**) of *Mt2* were increased in cells with *Mt2* overexpression. Furthermore, cell proliferation assay showed that *Mt2* overexpression promoted cell proliferation and the expression of genes associated with cell proliferation (**C, D**). ★ indicates significant differences (*p* < 0.05). ★★ indicates extremely significant differences (*p* < 0.01).

**Figure 7:**
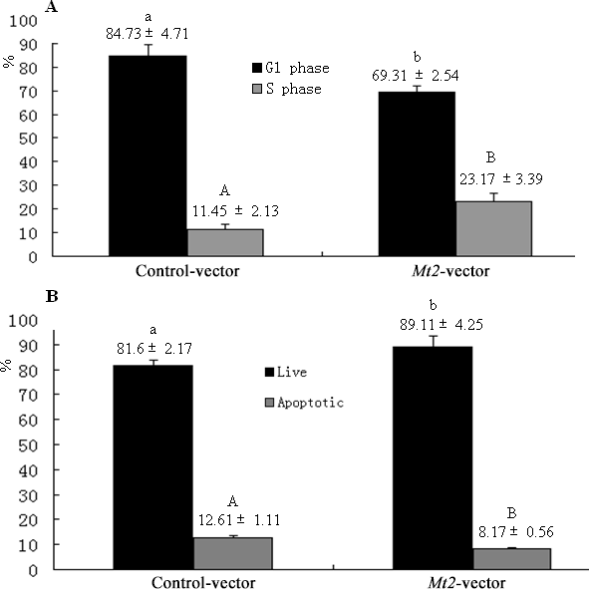
Cell cycle and apoptosis after *Mt2* overexpression in GC-1 spg cells The percentage of cells in the G1 phase was decreased, and the percentage of cells in the S phase was increased with *Mt2* overexpression (**A**). Meanwhile, the percentage of apoptotic cells was decreased when GC-1 spg cells were transfected with *Mt2* plasmid (**B**). Different letters indicate significant differences (*p* < 0.05). In apoptosis assay, the percentage of apoptotic cells included both early and late apoptosis.

The GC-1 spg cells were transfected with *Mt2* expression plasmid (pCMV6-*Mt2*) to investigate whether *Mt2* is an essential mediator of the effect of melatonin on the proliferation of GC-1 spg cells. The results showed that the expression of *Mt2* was remarkably increased after transfection with pCMV6-*Mt2* (Figure 6A, 6B). The CCK8 assay demonstrated that cell proliferation of the *Mt2*-transfected cells grew more rapidly than the control cells did (Figure 6C). Real-time PCR revealed that the expression of genes associated with cell proliferation was also increased (Figure 6D). Flow cytometric analyses showed that the percentage of GC-1 spg cells in the S phase was higher than that of the control cells because of the overexpression of *Mt2* (Figure 7A). Double-staining with Annexin V and propidium iodide showed that *Mt2* decreased the percentage of apoptotic cells relative to that of the control groups (Figure 7B).

**Figure 8:**
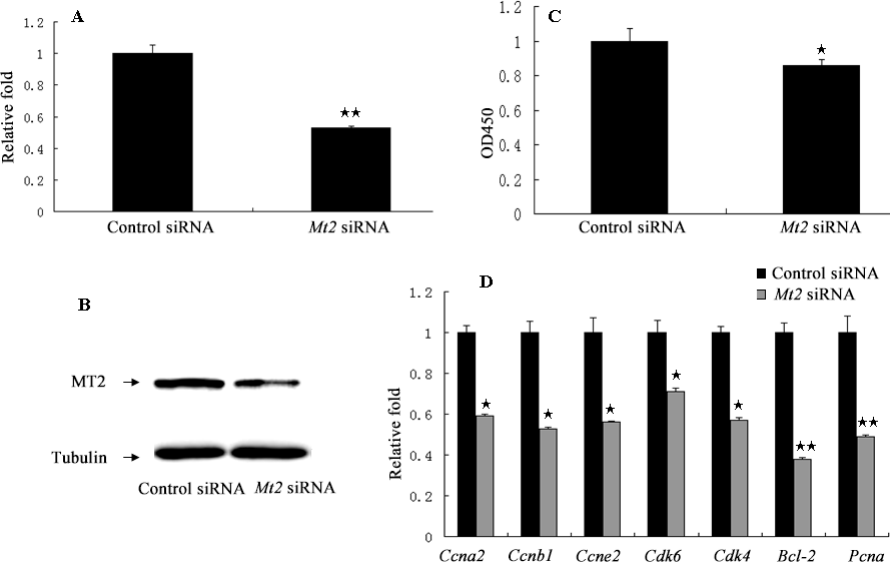
The cell proliferation after *Mt2* knockdown in GC-1 spg cells GC-1 spg cells were transfected with *Mt2* siRNA. Both mRNA (**A**) and protein (**B**) of *Mt2* were decreased in cells with *Mt2* siRNA. Cell proliferation assay showed that *Mt2* knockdown inhibited cell proliferation (**C**). The expression of genes involved in cell proliferation was decreased with *Mt2* siRNA (**D**). ★ indicates significant differences (*p* < 0.05). ★★ indicates extremely significant differences (*p* < 0.01).

**Figure 9:**
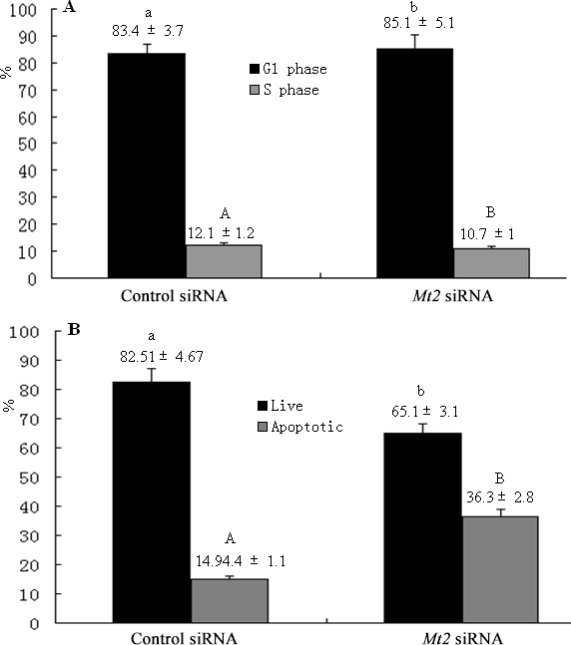
The cell cycle and apoptosis after *Mt2* knockdown in GC-1 spg cells The percentage of cells in the G1 phase was increased, and the percentage of cells in the S phase was decreased with *Mt2* siRNA (**A**). Meanwhile, the percentage of apoptotic cells was increased after *Mt2* knockdown in cells (**B**). Different letters indicate significant differences (*p* < 0.05).

A complementation experiment was then performed. GC-1 spg cells were transfected with *Mt2*-specific siRNA or a nonspecific siRNA. The transfection significantly decreased the *Mt2* expression in GC-1 spg cells transfected with *Mt2* siRNA than in the cells in the control siRNA group (Figure 8A, 8B). CCK8 assay demonstrated that the proliferation of GC-1spg cells was significantly inhibited after *Mt2* siRNA transfection was performed (Figure 8C). The *Mt2* knockdown in GC-1 spg cells significantly downregulated the expression of genes associated with cell proliferation (Figure 8D). Furthermore, *Mt2* knockdown in GC-1 spg cells decreased the percentage of cells in the S phase (Figure 9A) and increased the percentage of apoptotic cells relative to those in the control group (Figure 9B).

**Figure 10:**
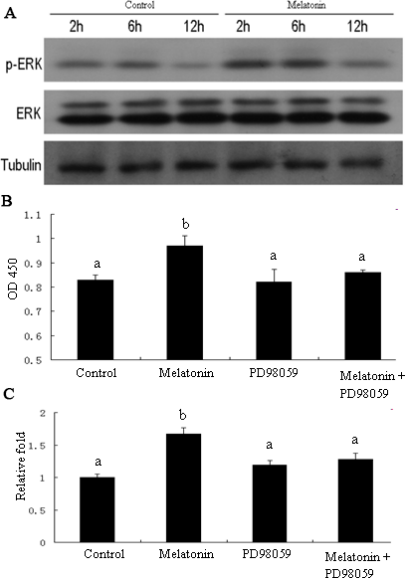
ERK1/2 signaling pathway involved in melatonin-regulated *Mt2* expression and cell proliferation in GC-1 spg cell GC-1 spg cells were treated with melatonin of 10nM for 2 hrs, 6 hrs, and 12 hrs. The level of ERK1/2 phosphorylation was increased after melatonin treatment and detected by Western blot (**A**). Cells were treated ERK1/2 signaling inhibitor (PD98059, S1805, Beyotime, 30μM) for 2 hrs, then followed by melatonin treatment for 24 hrs. PD98059 inhibited the melatonin-induced proliferation of GC-1 spg cells (**B**). The qRT-PCR showed that *Mt2* expression induced by melatonin was also decreased after PD98059 treatment (**C**). Different letters indicate significant differences (*p* < 0.05).

**Figure 11:**
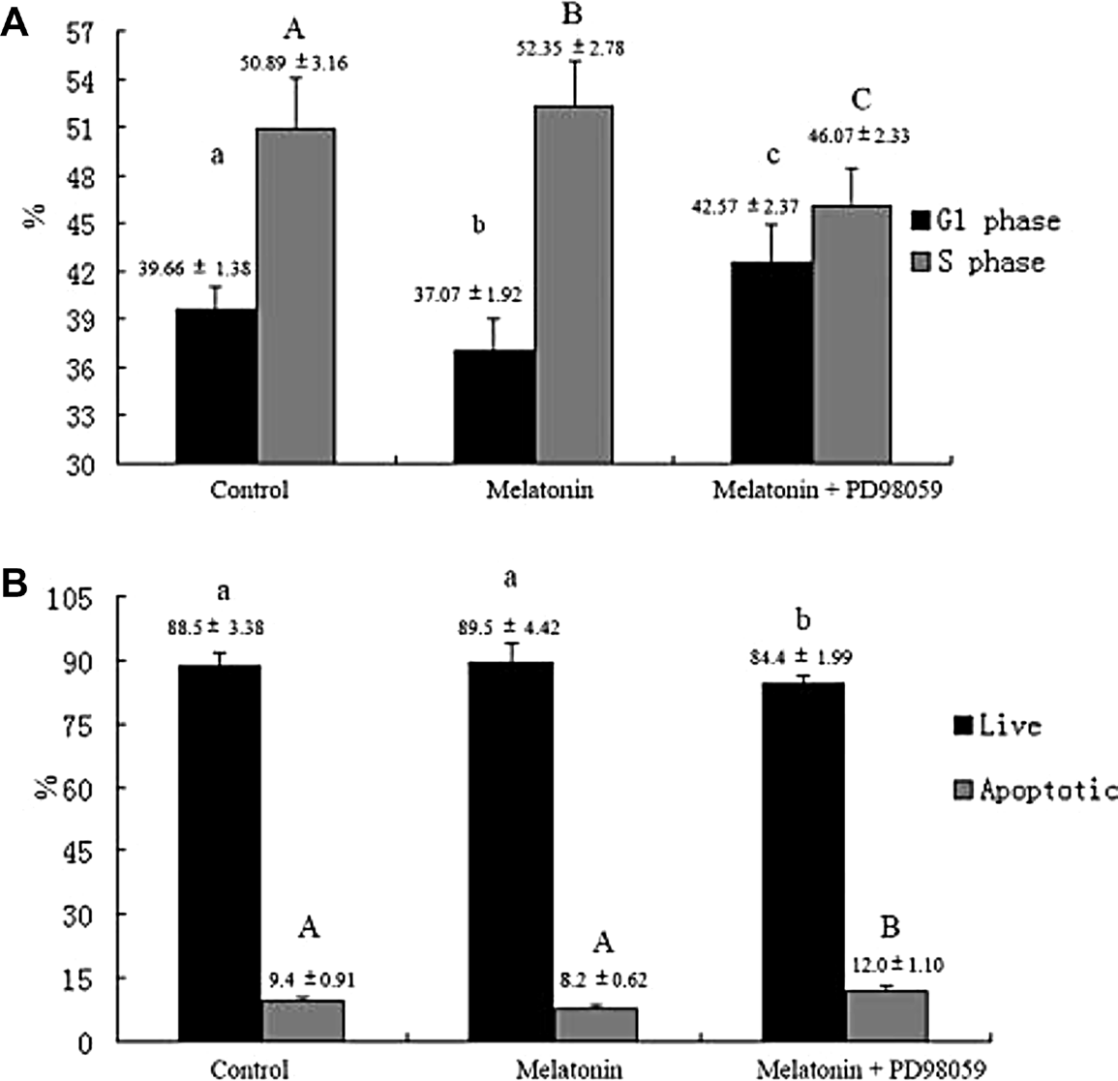
ERK1/2 signaling pathway involved in melatonin-regulated cell cycle and apoptosis in GC-1 spg cells PD98059 decreased the percentage of cells in the S phase and increased the percentage of cells in the G1 phase (**A**). Furthermore, PD98059 increased the percentage of apoptotic cells (**B**). Different letters indicate significant differences (*p* < 0.05).

Mitogen-activated protein kinases (MAPKs) are implicated in cell growth. Thus, we investigated whether melatonin treatment affects MAPK signaling in melatonin-induced proliferation of GC-1 spg cells. Results showed that cell treatment with melatonin at different time points (2, 6, and 12 h) activated the phosphorylation of ERK1/2 (Figure 10A). We then investigated whether MAPK signaling is involved in melatonin-induced proliferation of GC-1 spg cells. The cells were treated with ERK1/2 inhibitor (PD098059) and subjected to melatonin treatment. Results revealed that ERK1/2 inhibitor significantly inhibited the melatonin-induced proliferation in GC-1 spg cells (Figure 10B), downregulated the expression of *Mt2* (Figure 10C), decreased the percentage of cells in the S phase (Figure 11A), and increased the number of apoptotic cells (Figure 11B). Thus, melatonin mediated the upregulation of *Mt2* and subsequently increased the proliferation of GC-1spg cells by activating the MAPK signaling cascade.

## DISCUSSION

Germ cells in spermatogenesis contain the same genome, but only some genes are used in each stage of spermatogenesis. Some of these genes are expressed when necessary or stimulated by other factors [25]. Spermatogenesis can be elucidated by determining how genes are controlled and expressed at the exact time and what genes function in specific stages. Different methods, such as RT-PCR, Northern blot, and *in situ* hybridization, have been employed to detect genes [26–28], but these methods can focus on some genes at a time. As such, advanced approaches have been utilized to detect gene expression profiles and analyze gene expression with high throughput [29, 30]. For instance, cDNA microarray technology has been applied to investigate gene expression profiles in mouse spermatogenic cells in different stages of spermatogenesis [18]. Numerous genes have been detected. 181 genes in primitive type A spermatogonia, 256 genes in type B spermatogonia, 221 genes in preleptotene spermatocytes, 160 genes in pachytene spermatocytes, 141 genes in round spermatids, and 126 genes in elongating spermatids; these findings provide additional information for spermatogenesis research [18]. RNA sequencing technology has also revealed that gene expression patterns have significantly differed between mouse type B spermatogonia and primary spermatocytes; therefore, the basic molecular mechanism of early spermatogenesis is strongly related to the dynamics of cell junction [19].

In the present study, GC-1 spg cells were used as a model to investigate the role of melatonin in spermatogenesis. The physiological concentration of melatonin significantly promoted the proliferation of GC-1 spg cells. Melatonin can also enhance the differentiation of spermatogonial stem cells into haploid germ cells in sheep testis *in vitro* by activating melatonin membrane receptors and spermatogenesis-related gene expression [17]. Accumulated studies revealed that melatonin processes a beneficial function on human sperm motility [31], ram and pig sperm quality [32, 33]. Melatonin also improves the quality of thawed bovine serum by decreasing lipid peroxidation and enhancing the activity of antioxidant enzymes [34]. Intraperitoneal injection with 50 mg/kg melatonin significantly decreased levels of oxidative stress enzymes and lipid peroxidation in a rat model of testicular torsion induced by ischemia-reperfusion (I/R) injury [35]. Anticancer drugs can cause testicular toxicity, melatonin administration helped protect against drug-induced testicular toxicity [36, 37]. The protective roles of melatonin in testicular damage suggest that it may have clinical applications as a free radical scavenger and indirect antioxidant in the treatment of testicular damage.

Our previous study showed that melatonin can influence the testicular functions by regulating the differential expression of microRNAs in mouse testis [11]. Furthermore, melatonin can promote the proliferation of GC-1 spg cells by downregulating the expression of miR-16 and thus indicated that melatonin may regulate spermatogenesis [11]. To further explore the mechanism of melatonin in regulating spermatogenesis, we performed transcriptome sequencing and provided an in-depth analysis of the transcriptome of GC-1 spg cells exposed to melatonin. GO and KEGG analyses showed that differentially expressed genes, including *Ccne1, Ercc1,* and *Mt2*, were implicated in cell proliferation. *Ccne1* is a positive regulator of G1/S phase transition and important factor in cell cycle [38]. The knockdown of *Ccne1* inhibits cell growth [39]. *Ccne1* is overexpressed in most tumor cells [40]. The antitumor effects of melatonin are associated with the downregulated *Ccne1* expression [41]. *Ercc1* is essential for the repair of nucleotide excision and DNA double-strand breaks [42]. The disruption of mouse *Ercc1* causes cell cycle abnormalities in the liver and kidney before weaning [43]. Interestingly, *Ercc1* is expressed in the testis at high levels, and the knockout of *Ercc1* in mice increases the level of DNA strand breaks; oxidative DNA damage is also observed in *Ercc1*-deficient testis, and increased apoptosis is noted in male germ cells [44]. Melatonin elicits beneficial effects on the testis by preventing oxidative stress-evoked DNA damage in human spermatozoa and alleviating Cd-induced cellular stress and germ cell apoptosis, which may be associated with the melatonin-regulated expression of *Ercc1* [45]. As another important upregulated gene, *Mt2* is a member of metal-binding proteins, such as metallothioneins (Mts) [46]. MTs are divided into four subgroups that show tissue-specific expression and various functions. *Mt* genes are also actively expressed in male germ cells of mice [47] and rats [48]. The *Mt1* and *Mt2* expression levels in the testes from sexually mature adults are 10-fold higher than those in the livers of control adults [49]. The increased expression of *Mt1* and *Mt2* induced by melatonin in the testis mainly sequesters nonessential toxic heavy metals [50, 51]. However, studies have yet to provide evidence supporting high *Mt2* levels in GC-1 spg cells associated with melatonin-promoted cell proliferation. The overexpression of *Mt2* promoted the proliferation of GC-1 spg cells, whereas the knockdown of *Mt2* significantly suppressed the proliferation of GC-1 spg cells and induced their apoptosis. These results indicated that *Mt2* might be involved in melatonin-induced cell proliferation. The elevated level of *Mts* has been found in rapidly proliferating cells and various tumors [52–54]. In the analysis of the cycle of human colonic cancer cells, the maximum concentration of *Mt* is in successive late G phases and G1/S transition [53]. However, silencing *Mt2* in breast cancer cells inhibits the progression the cell cycle from G1 phase to S phase [55]. Therefore, *Mt2* could modulate cell proliferation by regulating cell cycle progression.

ERK1/2 signaling pathway mediates various cellular behaviors, including cell growth and apoptosis, in response to extracellular stimulation [56, 57]. The ERK1/2 pathway may also control the actions of melatonin [58, 59]. To identify whether ERK1/2 pathway is involved in the melatonin-promoted proliferation of GC-1 spg cells, we examined the ERK1/2 signaling pathway through Western blot and found that melatonin activated the phosphorylation of ERK1/2 in GC-1 spg cells. However, the suppression of ERK1/2 pathway significantly inhibited the proliferation of GC-1 spg cells and downregulated the expression of *Mt2* induced by melatonin.

In summary, our study revealed the expression profile of genes in GC-1 spg cells stimulated by melatonin. Melatonin could induce the differential expression of genes in GC-1 spg cells. The melatonin-promoted proliferation of GC-1 spg cells might be associated with the upregulation of genes involved in cell proliferation. The functional characterization of genes demonstrated that *Mt2* was regulated by melatonin and mediated melatonin-induced cell proliferation. The melatonin-induced expression of *Mt2* was mediated by the ERK1/2 pathway during GC-1 spg cell proliferation. These findings revealed that melatonin might be essential for spermatogenesis. However, the effects of melatonin on germ cell differentiation have yet to be observed because of the absence of a melatonin null model. Hence, further studies using genetically inactivated mice may help clarify their effects on this process.

## MATERIALS AND METHODS

### Cell culture

The mouse-derived spermatogonial cell line, GC-1 spg cells, (ATCC, VA, USA) were cultured under controlled conditions (37°C, 5% CO_2_) and grown in Roswell Park Memorial Institute (RPMI-1640, Hyclone, MA, USA ) medium supplemented with 10% FBS (Gibco, CA, USA) and antibiotics (penicillin 100 IU/mL and streptomycin 100 µg/mL, Hyclone, MA, USA ). At 80% confluence, the cells were subcultured with 0.05% trypsin (Gibco, CA, USA) for further experiments.

### Cell proliferation assay

The proliferation of GC-1 spg cells were evaluated by cell counting kit 8 (CCK8) assay (Beyotime Biotech, Haimen, China). In brief, these cells were seeded in 96-well plates at a density of 1 × 10^4^ cells per well with the complete growth medium. 12 hrs after seeding, cells were serum starved for 12 hrs then different melatonin concentrations in complete growth medium were added for another 24 hrs treatment. At the end of treatment time, 10 µL of the CCK8 solution was added to each well and incubated at 37°C for 2 hrs. The number of cells was identified in triplicates by determining the absorbance in a 96-well microplate reader (ELx800, BioTek, Inc., IL, USA) at a wavelength of 450 nm (OD450).

### High-resolution mRNA sequencing and data processing

GC-1 spg cells were seeded in a 6-well plate at a density of 1 × 10^6^. Cells were serum free starved for 12 hrs when they reached 50% confluence. Then 10 nM melatonin in complete growth medium was replaced for another 24 hrs treatment. Total RNA was isolated by using TRIzol reagent according to the manufacturer’s instructions (Invitrogen, CA, USA). To establish a paired-end RNA-seq library for transcriptome analysis, we isolated 10 µg of total poly(A) mRNA by using Sera-Mag Magnetic Oligo(dT) Beads (Sigma, MO, USA), broke the isolated mRNA into short fragments (Multiskan Spectrum; Thermo Scientific Multiskan), and added adapters. An appropriate size of the fragments was selected and amplified through PCR. Fragmentation buffer was added to divide the mRNA into short fragments. These short fragments were used as templates and a random hexamer primer was utilized to synthesize the first-strand cDNA. The second-strand cDNA was also synthesized with 20 µL of the second-strand buffer (Invitrogen, CA, USA), 10 mM of dNTP Mix, 5 U/µL of RNaseH, and 10 U/µL of DNA polymerase I. The short fragments were purified with a Qiaquick PCR purification kit (Qiagen, Dusseldorf, Germany) and resolved with EB buffer for the end reparation and poly(A) addition. The short fragments were then connected with sequencing adapters. After agarose gel electrophoresis was conducted, suitable fragments as templates were selected for PCR amplification. The paired-end (PE) RNA-seq libraries were prepared and sequenced on an Illumina HiSeq™ 2500 platform according to the manufacturer’s protocols. Data analysis and base calling were performed with Illumina instrument software. High-throughput sequencing was carried out with Beijing origene science and technology corp., ltd (Beijing, China). Differential expression (DE) results were based on the fold changes of the expression levels (*p* < 0.05) obtained by using Cufflinks (version 2.0.2). The DE genes were clustered through COG analysis by performing Fisher’s and chi-square tests.

The unigenes were used for BLASTX and annotation against protein databases, including nonredundant (nr), SwissPort, COG, KEGG, and GO, with a cutoff E-value of 0.00001. After the GO annotation for each unigene was obtained, GO functional classification for all unigenes was conducted and the distribution of gene functions was elucidated in WEGO. The KEGG pathway annotation was performed using blastall software instead of the KEGG database.

### Real-time quantitative PCR

GC-1 spg cells were seeded in a 12-well plate at a density of 5 × 10^5^. Cells were serum free starved for 12 hrs when they reached 50% confluence. Then 10 nM melatonin in complete growth medium was replaced for another 24 hrs treatment. Total RNA was extracted using a Total Cell RNA isolation kit (TIANGEN, Beijing, China). First-strand cDNA was synthesized using a PrimeScript first-strand cDNA synthesis kit (TaKaRa Biotechnology, Dalian, China) according to the manufacturer’s protocol. Real-time quantitative PCR was performed in triplicate in 96-well plates in an ABI Prism 7500 sequence detection system (Applied Biosystems, CA, USA). Reverse transcription was performed at 25°C for 10 min, 42°C for 20 min, and 85°C for 5 min. PCR was performed at 95°C for 5 min, followed by 40 cycles of 95°C for 15 s and 59°C for 30 s. Relative expression was normalized to *Hprt* RNA via ΔΔ*C*_t_ method. The sequences of the primers used for qRT-PCR are shown in Table 1.

**Table 1:** List of primers used for qRT-PCR

**Primer name**	**Sequence 5**ʹ**-3**ʹ
*Bbc3-F*	CCCAGAAATGGAGCCCAACT
*Bbc3-R*	CAAGGCTGGCAGTCCAGTAT
*Kcnn4-F*	CAACAAGGCGGAGAAACACG
*Kcnn4-R*	GTCCTTCCTTCGAGTGTGCT
*Mt2-F*	TCCATCACGCTCCTAGAACTC
*Mt2-R*	CAGGATCCATCGGAGGCACA
*Ercc1-F*	TAGCATCATCGTGAGCCCG
*Ercc1-R*	GCCCAGCACATAATCGGGAA
*Ccne1-F*	AGGCGAGGATGAGAGCAGTT
*Ccne1-R*	GGTGCAACTTTGGAGGGTAGA
*Hprt-F*	TCCTCCTCAGACCGCTTTT
*Hprt-R*	CCTGGTTCATCATCGCTAATC
*Ccna2-F*	CCTACCTCAAAGCGCCACAA
*Ccna2-R*	CTCCGAGCGACTCCTGTTTC
*Ccnb1-F*	TTGTGTGCCCAAGAAGATGCT
*Ccnb1-R*	GTACATCTCCTCATATTTGCTTGCA
*Ccne2-F*	CTGCTGCCGCCTTATGTCAT
*Ccne2-R*	TACACACTGGTGACAGCTGC
*Cdk6-F*	TCTCACAGAGTAGTGCATCGT
*Cdk6-R*	CGAGGTAAGGGCCATCTGAAAA
*Cdk4-F*	TTGTGCAGGTAGGAGTGCTG
*Cdk4-R*	TGCCAGAGATGGAGGAGTCT
*Bcl-2-F*	TGGAGAGCGTCAACAGGGAGA
*Bcl-2-R*	GCCAGGAGAAATCAAACAGAGGT
*Pcna-F*	TAAAGAAGAGGAGGCGGTAA
*Pcna-R*	TAAGTGTCCCATGTCAGCAA

### Western blot analysis

Protein lysates were prepared with lysis buffer supplemented with 1 mM PMSF (Beyotime Biotech, Haimen, China). Normalized proteins (20 μg) from each sample were electrophoresed with SDS-PAGE gels. Western blot analysis was performed with primary antibodies specific for MT2 (BS7352, Bioworld, Nanjing, China), ERK1/2 (BS1112, Bioworld, Nanjing, China), p-ERK1/2 (BS4621, Bioworld, Nanjing, China), or tubulin (AF0001, Beyotime Biotech, Haimen, China). After the reaction with HRP-conjugated goat anti-rabbit secondary antibody (A0208, Beyotime Biotech, Haimen, China) occurred, the membranes were detected with BeyoECL Plus kit (Beyotime Biotech, Haimen, China).

### Cell cycle analysis

Flow cytometric analyses were performed to define the cell cycle distribution. In brief, GC-1 spg cells were seeded in a 12-well plate at a density of 5 × 10^5^. Cells were serum free starved for 12 hrs when they reached 50% confluence. Then 10nM melatonin in complete growth medium was replaced for another 24 hrs treatment. Cells were harvested with trypsin and fixed with 70% ethanol at 4°C for 12 hrs. After ethanol was removed through centrifugation, the cells were washed with PBS and stained with 50 μg/mL propidium iodide and 50 μg/mL RNase A (Beyotime Biotech, Haimen, China) at 37°C for 30 min. Cell cycle distribution was analyzed by using a Cytomics FC500 flow cytometer (Beckman Coulter, Inc., CA, USA).

### Apoptosis assay

Apoptosis was assessed by using an Annexin V-FITC apoptosis detection kit (Beyotime Biotech, Haimen, China). GC-1 spg cells were seeded in a 12-well plate at a density of 5 × 10^5^. When cells occupied 50% confluence, 12 hrs serum free starvation followed by 24 hrs melatonin of 10nM with complete growth medium treatment was performed. Cells were collected by centrifuge, resuspended in 100 μL of buffer with 5 μL of Annexin V and 1 μL of propidium iodide, and incubated for 15 min at 25°C in the dark. Buffer (400 μL) was added to obtain the final volume of 500 μL. The cells were immediately analyzed with a Cytomics FC500 flow cytometer (Beckman Coulter Inc., CA, USA).

### Plasmids, siRNAs, and transfection

pCMV6-empty plasmid vector (OriGene Technologies, MD, USA) and pCMV6 carrying the full-length mouse *Mt2* cDNA (gene ID: 17750) were prepared by using a ClonExpress II one-step cloning kit (Vzayme, Nanjing, China). The sequences of siRNAs (RiboBio, Guangzhou, China) for *Mt2* were used in the knockdown experiments. For the transient transfection of plasmids (200ng for 96-well plate and 1500ng for 12-well plate) or siRNAs (5pmol for 96-well plate and 40 pmol for 12-well plate), GC-1 spg cells were cultured in 96- or 12-well plates with complete growth medium until 70% confluence was reached. The cells were washed once with PBS and then transfected in a serum-free RPMI-1640 medium by using Lipofectamine 2000 (Invitrogen, CA, USA) according to the manufacturer’s instructions. After 12 hrs transfection, medium was changed to complete growth medium for another 24 hrs incubation.

### Statistical analysis

Experiments were performed at least thrice. Differences between groups were compared through one-way ANOVA, followed by Dunnett’s multiple range or chi-square test for multiple comparisons in SPSS. Results were expressed as mean ± standard deviation. A *p*-value of < 0.05 was considered statistically significant.

## SUPPLEMENTARY MATERIALS TABLE



## Abbreviations

*Mt2*: metallothionein-2
ERK1/2: extracellular-signal-regulated kinase 1/2
KEGG: Kyoto Encyclopedia of Genes and Genomes
GO: Gene ontology
CCK8: cell counting kit 8
siRNA: small interfering RNA
MAPK: mitogen-activated protein kinase
*Ccne1*: cyclin E1
*Ercc1*: ERCC excision repair 1
