# Supplementary material for: Melatonin promotes the proliferation of GC-1 spg cells by inducing metallothionein-2 expression through ERK1/2 signaling pathway activation
Source: Oncotarget. 2017 Aug 7;8(39):65627–41. doi: 10.18632/oncotarget.20019 (PMC5630359; doi:10.18632/oncotarget.20019)
Supplement: Supplementary file 1 [file oncotarget-08-65627-s001.pdf]

Melatonin promotes the proliferation of GC-1 spg cells by inducing metallothionein-2 expression through ERK1/2 signaling pathway activation

SUPPLEMENTARY MATERIALS

Supplementary Table 1: Differentially expressed mRNAs between control (C) and melatonin-treated (M) group in GC-1 spg cells, showing as the fold changes of M/C expression values

Genes (Upregulation)	Fold change	Genes (Downregulation)	Fold change
Ccne1	1.3	*Bbc3*	0.8
Ercc1	2.2	*Prrg2*	0.3
Them4	2.1	*Lamc2*	0.5
Coq3	2.2	*Psd4*	0.2
Mettl10	2.3	*Tceanc2*	0.0
Mt2	2.0	*Ptprf*	0.5
Traf3ip1	2.3	*Dbn1*	0.5
Zfp580	2.2	*Mtg1*	0.4
Ttc26	2.2	*Ppp1r3f*	0.5
Gm10184	2.1	*Ccr4*	0.5
Gm26870	2.9	*Olfr368*	0.5
		Klhdc1	0.4
		Ccdc171	0.5
		Kcnn4	0.8
		Gm15298	0.5
		4933407K13Rik	0.3
		RP23-246J18.5	0.4
